# Sleep – the guarantee of health! Does the environmental perception characteristics of urban residential areas affect residents' sleep quality?

**DOI:** 10.3389/fpubh.2022.1017790

**Published:** 2023-01-18

**Authors:** Xun Zhu, Ming Gao, Xinting Cheng, Wei Zhao

**Affiliations:** ^1^School of Architecture, Harbin Institute of Technology, Harbin, China; ^2^Key Laboratory of Cold Region Urban and Rural Human Settlement Environment Science and Technology, Ministry of Industry and Information Technology, Harbin, China

**Keywords:** environmental perception, sleep quality, positive mental health, physical environment, wellbeing, social environment

## Abstract

A complex urban living environment and residents' sleep quality are intrinsically linked. Nonetheless, there is little evidence that the residential environment affects sleep quality. Based on the results of subjective questionnaires, this study uses the multiple regression combined with mediation analysis to construct a mechanical model of the impact of urban residential environmental perception characteristics on residents' sleep quality. Moreover, the differences among the influence intensities of the significant factors are compared and the results show that (1) in low-density environments (FAR < 2) and lower floors (4–6), residents sleep longer and have better sleep quality; (2) the environmental quality and service facilities of the physical environment and the sense of safety in the social environment have a significant impact on residents' sleep quality; and (3) the mental health of residents play a significant intermediary role in the relationship between social environment and sleep quality, with the highest effect accounting for 33.88%. The influence mechanisms of various environmental factors in a residential area on sleep quality were revealed and a more refined design basis for a healthy urban living environment, community renewal, and renovation was provided.

## Graphical Abstract

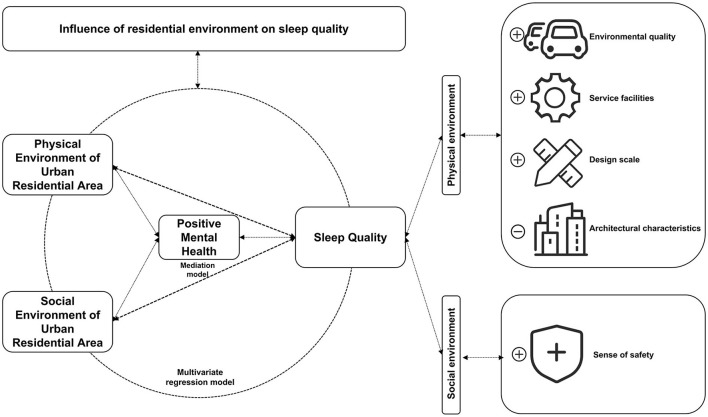

## 1. Introduction

Good sleep quality can improve human health and wellbeing (1, 2). However, urban residents are vulnerable to the impact of the urban residential environments, which will inevitably affect their sleep level. For urban dwellers, sleep is essential not only for their physical and mental health but also for their work, study, and life. Sleep quality significantly impacts workplace accidents, productivity, job satisfaction, and wellbeing (3–5). Sleep is essential for the effective cognitive and emotional processing of the people (6). However, in modern cities with rapid technological growth, it is difficult for urban residents to sleep well (7). In addition, insufficient sleep has been associated with many health benefits, including cardiovascular abnormalities (8), hypertension (9), diabetes (10), and a higher risk of death (11). The incidence of sleep disorders among college students in China is close to 20% (12), and the overall prevalence of sleep deficiency among the elderly has reached 35.9% (13). Moreover, the age-adjusted rate of insomnia in the United States is 18.8% (14). Concerning the high incidence rate of sleep and its harmful effects, sleep has gradually become a noticeable public health problem (15).

To improve the sleep quality of urban residents, it is vital to identify the residential environmental factors that lead to sleep disorders. The factors affecting sleep quality were confirmed by exposure to the urban physical environment. The most common impact factors are ambient noise (16) or the built environment [e.g., urbanization and residential density (17), roads (18), and recreational use (19)]. Other physical environmental exposures include air pollution (20, 21), thermal comfort (22), outdoor lighting at night (23, 24), residential green space (25, 26), and mobile phone radiofrequency electromagnetic field (RF-EMF) (27). The urban residential environment is complex and unique, and the characteristics of residents' sleep quality in different residential environments may differ. However, the internal mechanism of the impact of a residential environment on residents' sleep quality still needs to be determined.

Among the psychosocial and environmental factors in the ecosystem (28), neighborhoods are potential factors for good sleep and insomnia prevention. Adverse neighborhood and social environment (low security and social cohesion) are related to high incidences of short sleep and insomnia (29). Residents in daily life can perceive the environmental characteristics of these urban settlements, such as the road width of the residential area, the abundance of vegetation, and the quality-of-service facilities. They also include the social environmental characteristics formed by the residential area, such as neighborhood trust, social capital, and cohesion as perceived by the residents (30). In addition, a global analysis result of six countries shows that perceived neighborhood safety is negatively related to insomnia symptoms and poor sleep quality (31). Therefore, perception of the environment is also essential (32). The perceived physical environment has a direct impact on sleep quality. However, residents' perceived social environment may also affect sleep quality. Therefore, it is necessary to explore a multilevel environment, which will help fully understand the impact of environment on the sleep quality of urban residents.

In addition, the psychosocial determinants of insomnia have been further confirmed (33, 34). They are believed to play a role in developing sleep disorders (35). Some existing studies have found that better mental health (for example, reducing anxiety and depression) can improve the quality of life and sleep of the public (36, 37). Mental health is the experience of residents' satisfaction and subjective wellbeing, reflecting the positive emotions, interpersonal satisfaction, and positive psychological functions of the individual's mental health about the environment (38). Therefore, mental health is one of the risk factors for sleep quality (1). Whether mental health has an indirect role in the impact of environment on sleep quality still needs to be explored. It is necessary to investigate how mental health are related to sleep (39).

**Figure 1:**
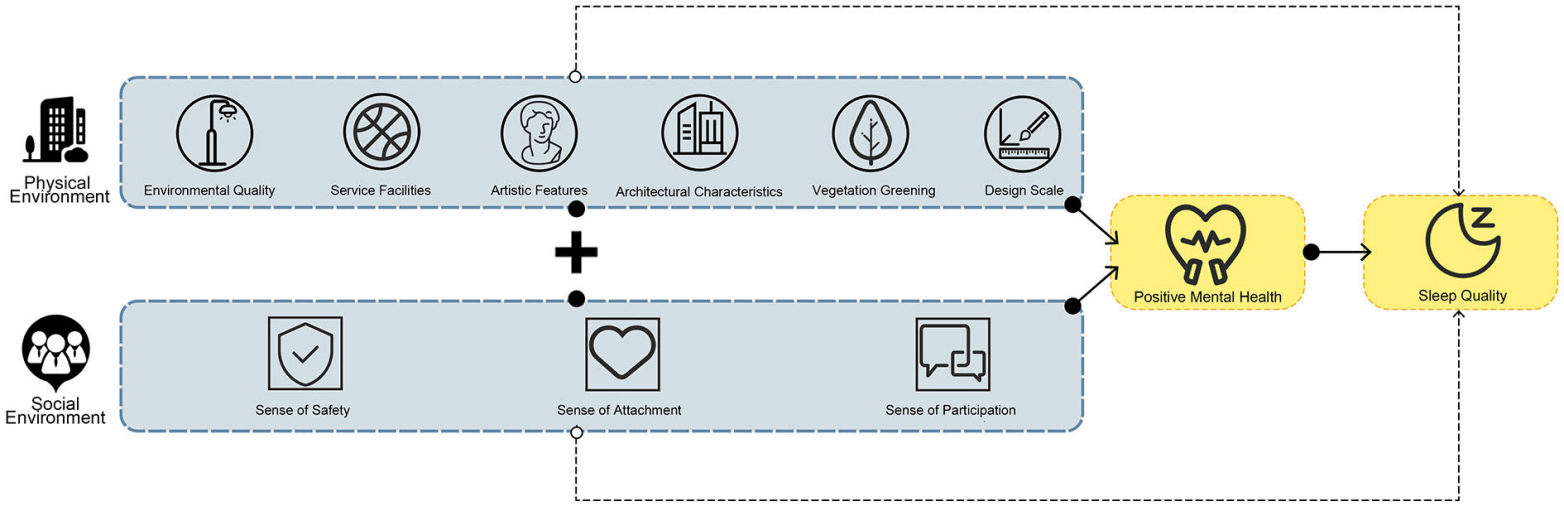
Research content framework.

This study aims to clarify the relationship between the urban residential environment and residents' sleep quality to find out the potential spatial environmental factors that affect sleep quality, examine the mediating effect of public mental health on the residential environment and sleep quality, as shown in Figure 1, and determine the impact of the environmental perception characteristics of urban residential areas on residents' sleep quality and provide theoretical guidance and optimization plans for the renewal and renovation of healthy cities and communities. In this work, we ask the following research questions:

- **Research question 1** (RQ1): Are there significant differences between residential density and sleep quality?
- **Research question 2** (RQ2): If the answer to research question 1 is positive, are there any residential environment characteristics associated with residents' sleep quality? Which environmental factors improve sleep quality and which reduce sleep quality?
- **Research question 3** (RQ3): If the answer to research question 2 is positive, does the mental health status of the residents affect the way the environment affects sleep quality?

## 2. Methods

### 2.1. Study sample

**Figure 2:**
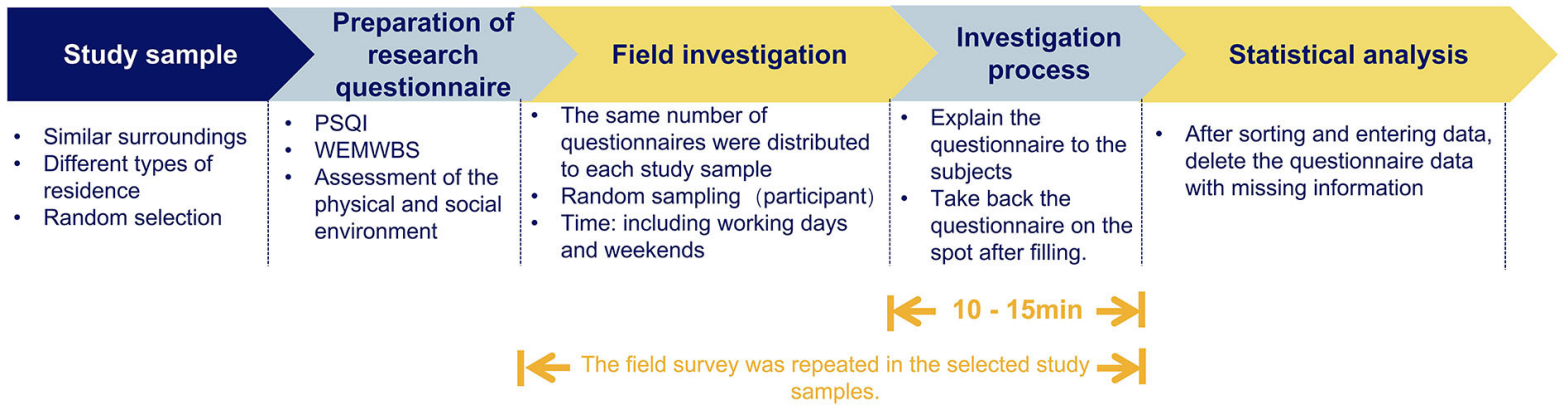
The procedure of the experiment.

Combined with the characteristics of urban areas, the research scope is on the main urban area of Jilin City, China. Its characteristics are mainly characterized by a relatively stable population and economic structure. The types of residential areas are diverse and adjacent to the same area. It is guaranteed that the surrounding area of the residential area has similar landuse conditions and that the residential area has a similar construction period and housing price range to ensure a more reasonable community economy. Seven typical residential areas were randomly selected as research samples, including the basic information about residential areas, plot ratio, and greening rate. The floor area ratio (FAR) that is often used in residential areas is the indicator to define the high- and low-densities of the residential areas (FAR > 2 is high density and FAR < 2 is low density). When arranging and entering data, excluding the missing questions, conflicting information, and incomplete information in the research questionnaire, a total of 500 samples were distributed and 438 valid samples were obtained. The procedure of the experiment is shown in Figure 2.

### 2.2. Ethics statement

In China, universities are not required to undertake a formal ethics review. However, an ethical approach is expected, and for this research, providing information and seeking permission from all participants to provide written informed consent forms as part of the study. The consent of each participant was obtained and the information filled in was only used for academic research.

### 2.3. Measures

#### 2.3.1. Residential environment

The residential environment is measured by physical and social dimensions as shown in Table 1. The physical environment mainly focuses on six aspects: environmental quality, architectural characteristics, service facilities, design scale, vegetation greening, and artistic features. The answer is set on a five scale from “very unsatisfactory to very agreeable.” It assigns a value from 1 to 5 and the respondents rate each item according to their perceptions. In terms of social environment, based on the social cohesion and trust scale proposed by Sampson (50), the perceived social welfare scale proposed by Volker (54), and the indicators related to social capital proposed by Buckner, the subitems were extracted and integrated. The district's social environment characteristics are divided into three categories: a sense of participation (55, 56), a sense of attachment (51), and a sense of safety. Answers are based on a 5-point Likert scale, ranging from 1 (very satisfied) to 5 (very dissatisfied).

**Table 1:** Residential physical environment and social environment assessment.

**Category**	**Features**	**Evaluation dimension**
Physical environment	Environmental quality	The degree of road cleanliness (40), the degree of vehicle aggregation, and the lighting conditions at night (41)
	Architectural characteristics	The degree of building enclosure, the degree of building transparency, and the degree of building continuity (42)
	Service facilities	Number of leisure facilities and the area of activity space (43)
	Design scale	Pedestrian road width (44, 45), building volume, and building (sunshine) spacing
	Vegetation greening	Green area (46), vegetation growth status, and vegetation collocation abundance (47)
	Artistic features	The richness of colors (48), the number of public art sculptures, and the degree of personalization of styles (49)
Social environment	Sense of participation	Neighborhood communication and trust (50)
	Sense of attachment	Place dependence and identity (51)
	Sense of safety	Environmental safety (52), communication safety, and traffic safety (53)

#### 2.3.2. Sleep quality

Sleep quality was measured using the Pittsburgh Sleep Quality Index (PSQI) (57, 58). It contains 19 items in seven dimensions. Each item was rated on a 5-point Likert scale to facilitate statistics and analysis, ranging from 1 (strongly disagree) to 5 (strongly agree), with higher scores indicating a higher PSQI. Furthermore, the amount table has been used in various populations and countries, and its reliability and validity have been well verified (59). In the subsequent analysis, to be able to elaborate on the impact of the environment on various aspects of sleep, we calculated each dimension of the PSQI separately.

#### 2.3.3. Mental health

The Warwick-Edinburgh Mental Well-Being Scale (WEMWBS) (60) was used to evaluate residents' mental health. WEMWBS measures mental health and interpersonal satisfaction from two levels of pleasure and happiness (60). This scale is widely used by researchers at home and abroad to directly measure mental health and has good reliability and validity (61). According to the research purpose, each item is set with five grades of “exactly like me, not quite like me, unclear, somewhat like me, completely me,” and assigns 1–5 points in turn.

### 2.4. Statistical analysis

On the basis of collecting sleep data questionnaires, Statistical Product and Service Solutions (SPSS) was used to conduct statistical analysis on the acquired data (62). The analysis of variance (ANOVA) was used to test the significant differences in self-rated sleep quality among related factors. The *t*-test was used to test the differences in residential density. It was used to test for significant differences at *p* < 0.01 and *p* < 0.05. The relationship between residents' sleep quality and the factors affecting the physical environment of the residential area, the relationship between sleep quality data and the influencing factors of the social environment of the residential area, and the relationship between mental health and sleep status were calculated by a Pearson correlation test. In addition, linear regression analysis was performed to examine the relationship between environment and sleep quality. Through the simple mediation model compiled by Hayes, using the process 3.3 plug-in in SPSS, the mediation effect test is carried out to explore the internal mechanism of the impact of the urban residential environment on sleep quality and decompose the complex influence path.

## 3. Results

### 3.1. Reliability and validity

In the research questionnaire, Cronbach's α coefficients of the urban residential environment assessment, positive mental health, and sleep quality scales were 0.80, 0.96, and 0.85, respectively, reflecting the high reliability of the scales in this study and the structure of the study. The Kaiser–Meyer–Olkin values of the Residential Environment Scale and the Positive Mental Health Dimension Scale are all >0.85. The Sleep Quality Scale is more significant than 0.75, and the Bartlett sphericity test is significant, indicating that the validity of the research questionnaire is effective.

### 3.2. Descriptive statistics

The physical and social environments of urban residential areas were analyzed using SPSS 25.0 statistical analysis software. The results of the statistical analysis are shown in Table 2. In the perceived physical environment, the public assessment scores for service facilities and design scale are relatively high, 4.4 and 4.2, respectively, and the lowest for artistic features is 3.11. As for the perception score of the social environment in residential areas, the highest security score is 4.1, followed by attachment and participation. In general, the residential area's perceived physical environment is slightly higher than the social environment score.

**Table 2:** Assessment scores of the physical and social environments of the urban residential area.

**Category**	**Features**	**Mean value**	**Standard deviation**
Physical environment	Environmental quality	3.844	0.878
	Vegetation greening	3.217	0.840
	Artistic features	3.110	0.871
	Architectural characteristics	3.511	0.822
	Design scale	4.264	0.870
	Service facilities	4.400	0.863
Social environment	Sense of participation	3.310	0.710
	Sense of attachment	3.419	0.756
	Sense of safety	4.172	0.707

### 3.3. Different results of different residential densities on sleep quality

Research question 1 (RQ1) focuses on the residents' sleep quality under the mapping of high- and low-density environments in urban residential areas. PSQI integrates all the dimensions of sleep and represents the overall sleep quality. Therefore, RQ1 can be answered by a *t*-test. It can be seen from Table 3 that the samples with different high and low densities showed significant significance (*p* < 0.05) for seven items: sleep quality, time to fall asleep, sleep efficiency, sleep disorder, drug hypnosis, disfunction, and sleeping time. Therefore, there are significant differences in sleep status under different-density environments in urban residential areas. According to the variance results, the sleep status in a high-density environment is generally lower than in a low-density residential environment. Compared with low-density residential areas, the sleep status of high-density residential areas (FAR > 2) is characterized by poor sleep quality, short sleeping time, long time to fall asleep, and poor sleep efficiency.

**Table 3:** Sleep status *t*-test analysis results under different residential densities.

**Definition**	**High and low density**		***t***	***p***
	**(mean** ± **standard deviation)**			
	**Low density (*****n*** = **155)**	**High density (*****n*** = **282)**		
Sleep quality	3.42 ± 0.61	3.20 ± 0.57	3.634	0.000^**^
Time to fall asleep	3.21 ± 0.99	2.87 ± 0.93	3.550	0.000^**^
Sleeping time	3.39 ± 0.96	3.08 ± 0.89	3.302	0.001^**^
Sleep efficiency	3.56 ± 0.95	3.24 ± 0.88	3.582	0.000^**^
Sleep disorder	2.60 ± 0.96	3.53 ± 0.79	−10.306	0.000^**^
Drug hypnosis	2.60 ± 0.86	3.39 ± 0.75	−10.028	0.000^**^
Disfunction	2.45 ± 0.88	3.24 ± 0.79	−9.605	0.000^**^

*p < 0.05,

** p < 0.01.

**When the confidence value (double test) is 0.01, the correlation is significant.

In addition, to further investigate the effect of the environment in which residents live on their sleep status, an ANOVA (known as a one-way ANOVA) was used to investigate the variability of the number of housing floors on seven indicators: sleep quality, sleeping time, sleep efficiency, sleep disorders, drug hypnosis, disfunction, and time to fall asleep. It can be seen from Table 4 that samples of different housing floors have significant effects on all dimensions of sleep quality (*p* < 0.05). Different floors also show significant differences in residents' sleep status. The results showed that the sleep status of residents on the 4th and 6th floors was relatively good. With the increase in the number of floors, the residents' sleep status shows a trend of rising first and then declining, especially for the public sleep status of high buildings (30 floors and above) and low floors (1–3 floors); in short, through the macro perspective of the density of residential areas and the different floors of living. Low-density residential areas, as well as those living on low floors (< 15 floors), perform better in sleep.

**Table 4:** Analysis of the variance results of different residential floors on residents' sleep status.

	**Number of residential floors**					***F***	***p***
	**(mean** ± **standard deviation)**						
	**1–3 (*****n*** = **75)**	**4–6 (*****n*** = **146)**	**7–15 (*****n*** = **80)**	**16–30 (*****n*** = **71)**	**Over 30 (*****n*** = **65)**		
Sleep quality	3.08 ± 0.56	3.47 ± 0.57	3.40 ± 0.59	3.20 ± 0.55	3.03 ± 0.59	10.562	0.000^**^
Time to fall asleep	2.15 ± 0.77	3.40 ± 0.79	3.69 ± 0.72	2.92 ± 0.69	2.26 ± 0.83	64.466	0.000^**^
Sleeping time	2.91 ± 0.95	3.39 ± 0.88	3.55 ± 0.94	2.97 ± 0.84	2.85 ± 0.80	10.604	0.000^**^
Sleep efficiency	3.05 ± 0.82	3.55 ± 0.95	3.55 ± 0.90	3.28 ± 0.86	3.08 ± 0.87	6.612	0.000^**^
Sleep disorder	3.89 ± 0.75	2.84 ± 0.86	2.50 ± 0.80	3.23 ± 0.61	4.05 ± 0.74	58.559	0.000^**^
Drug hypnosis	3.85 ± 0.73	2.75 ± 0.73	2.46 ± 0.62	3.10 ± 0.56	3.88 ± 0.74	69.862	0.000^**^
Disfunction	3.73 ± 0.68	2.55 ± 0.79	2.41 ± 0.74	3.01 ± 0.64	3.62 ± 0.76	56.051	0.000^**^

^*^p < 0.05,

** p < 0.01.

^**^When the confidence value (double test) is 0.01, the correlation is significant.

### 3.4. The influence of residential environment on sleep quality

#### 3.4.1. The relationship between physical environment characteristics and sleep quality

To answer research question 2 (RQ2), Pearson correlation analysis was used to study the relationship between urban living perceived physical environment and residents' sleep status. Table 5 shows no significant correlation between vegetation greening, artistic features, and architectural characteristics in sleep quality, sleeping time, and sleep efficiency. The rest of sleep status are significantly related to the environmental quality, design scale and service facilities in the physical environment of urban residential areas, which confirms that the physical environment of residential areas has a regulatory role in sleep status. In general, according to the correlation analysis results of the physical environment characteristics and sleep quality, the environmental characteristics, vegetation greening, artistic features, architectural characteristics, design scale, and service facilities are all related to sleep, among which the environmental quality and service facilities are more closely associated with public sleep. This is an initial answer to RQ2.

**Table 5:** Pearson correlation results of the physical environment and sleep.

	**Environmental quality**	**Vegetation greening**	**Artistic features**	**Architectural characteristics**	**Design scale**	**Service facilities**
Sleep quality	0.446^**^	0.036	−0.018	−0.063	0.231^**^	0.416^**^
Time to fall asleep	0.289^**^	0.168^**^	0.176^**^	0.133^**^	0.358^**^	0.312^**^
Sleeping time	0.340^**^	0.035	0.017	−0.021	0.175^**^	0.400^**^
Sleep efficiency	0.427^**^	−0.010	0.026	−0.015	0.180^**^	0.419^**^
Sleep disorder	−0.304^**^	−0.139^**^	−0.119^*^	−0.149^**^	−0.208^**^	−0.279^**^
Drug hypnosis	−0.280^**^	−0.154^**^	−0.177^**^	−0.191^**^	−0.206^**^	−0.296^**^
Disfunction	−0.236^**^	−0.224^**^	−0.158^**^	−0.170^**^	−0.191^**^	−0.239^**^

* p < 0.05,

** p < 0.01.

#### 3.4.2. The relationship between social environment characteristics and sleep quality

To further answer RQ2, as shown in Table 6, a Pearson correlation analysis was carried out between the residents' perceived social environment and sleep quality. Perceived social environment and sleep status showed a significant correlation, which further confirmed that the social environment in residential areas has a particular regulatory effect on residents' sleep status. The residents' sense of attachment and safety in the social environment is more strongly related to the public's sleep. Specifically, sleep quality, time to fall asleep, sleeping time, and sleep efficiency showed a significant positive relationship with the three aspects of the perceived social environment (*p* < 0.01).

**Table 6:** Pearson correlation results of social environment and sleep.

	**Sense of participation**	**Sense of attachment**	**Sense of safety**
Sleep quality	0.223^**^	0.275^**^	0.295^**^
Time to fall asleep	0.331^**^	0.333^**^	0.302^**^
Sleeping time	0.183^**^	0.219^**^	0.238^**^
Sleep efficiency	0.250^**^	0.305^**^	0.246^**^
Sleep disorder	−0.311^**^	−0.322^**^	−0.270^**^
Drug hypnosis	−0.355^**^	−0.343^**^	−0.286^**^
Disfunction	−0.342^**^	−0.340^**^	−0.276^**^

^*^p < 0.05,

** p < 0.01.

^**^When the confidence value (double test) is 0.01, the correlation is significant.

#### 3.4.3. Influence model of residents' sleep quality

With the help of the SPSS software, a multilayer multiple linear regression model was constructed for the dependent variable sleep quality. From the two previous sections, it can be confirmed that the residential area's physical and social environments significantly correlate with sleep quality. Step 1: According to the Pearson correlation analysis results of independent and dependent variables in the previous section, the relevant perceived physical environment characteristics are included to build model I. In Step 2, social environment features were added to build model II containing physical and social environment features. Beta in the model is the standard coefficient, the influence effect value. A positive value indicates that the variable and the residents' sleep quality level are positive influences. At the same time, multicollinearity was tested by a multiple regression linear analysis, and the variance expansion factor VIF values were all < 2, indicating that these independent variables did not cause this problem.

The results of model I are shown in Table 7, in which environmental quality (*B* = 0.333, *P* < 0.01) > service facilities (*B* = 0.281, *P* < 0.01) > design scale (*B* = 0.094, *P* < 0.05) have a significant impact degree and positive relationship and architectural characteristics (*B* = −0.083, *P* < 0.05) have a negative relationship. With the addition of social environment variables in model II, as shown in Table 8, the *R*^2^ increased to 0.296 after the model II adjustment (adjusted *R*^2^ was statistically significant, and *P* < 0.05). Among them, the influence degree of sense of safety was the most significant (*B* = 0.105, *P* < 0.05), and there was a positive relationship with sleep quality. The purpose is to explore the common influence of multiple dependent variables on the dependent variables through the multiple regression model and screen out the more significant influencing factors, namely, the environmental quality in the physical environment, the service facilities, and the sense of safety in the social environment—all answers to research question 2 (RQ2) of this pair.

**Table 7:** Results of multilevel regression equation model of sleep quality.

**Variable types**	**Variable**	**Model I**				**Model II**			
		**Standardized coefficients**	***t***	***p***	**VIF**	**Standardized coefficients**	***t***	***p***	**VIF**
		**Beta**				**Beta**			
Physical environment	Constant	–	11.080	0.000^**^	–	–	7.940	0.000^**^	–
	Vegetation greening	0.037	0.902	0.368	1.015	0.042	1.043	0.298	1.021
	Artistic features	−0.035	−0.855	0.393	1.006	−0.030	−0.734	0.463	1.010
	Environmental quality	0.333	7.615	0.000^**^	1.165	0.299	6.654	0.000^**^	1.248
	Design scale	0.094	2.211	0.028^*^	1.113	0.076	1.754	0.080	1.150
	Architectural characteristics	−0.083	−2.028	0.043^*^	1.013	−0.073	−1.802	0.072	1.019
	Service facilities	0.281	6.391	0.000^**^	1.175	0.251	5.616	0.000^**^	1.240
Social environment	Sense of participation					0.014	0.315	0.753	1.255
	Sense of attachment					0.053	1.135	0.257	1.341
	Sense of safety					0.105	2.271	0.024^*^	1.315

Dependent variable: sleep quality.

* p < 0.05,

** p < 0.01.

**Table 8:** Fitting degree of multiple regression model for children's sense of safety.

**Model**	***R* ^2^**	**Adjusted *R*^2^**	***F* change**	**Significant *F* change**
I	0.294	0.284	29.885	0.000
II	0.311	0.296	21.367	0.000

### 3.5. The relationship between mental health and sleep quality

#### 3.5.1. The direct influence of mental health on sleep quality

The purpose of research question 3 is to explore whether public mental health plays a mediating role in an urban environment and residents' sleep quality. Correlation analysis was used to study the relationships between positive mental health and sleep quality, time to fall asleep, sleeping time, sleep efficiency, sleep disorder, drug hypnosis, and disfunction. Table 9 shows that the level of mental health has a significant correlation with sleep status, and the maximum correlation coefficient between mental health and sleeping time is 0.847, showing a significance level of 0.01. In short, it preliminarily proves a significant correlation between mental health and the quality of public sleep.

**Table 9:** Pearson correlation results of positive mental health and sleep status.

	**Positive mental health**
Sleep quality	0.348^**^
Time to fall asleep	0.847^**^
Sleeping time	0.300^**^
Sleep efficiency	0.307^**^
Sleep disorder	−0.405^**^
Drug hypnosis	−0.445^**^
Disfunction	−0.415^**^

^*^p < 0.05,

** p < 0.01.

^**^When the confidence value (double test) is 0.01, the correlation is significant.

#### 3.5.2. The mediating effect of mental health on sleep quality

A Pearson correlation analysis found that mental health impacts residents' sleep quality. Therefore, after the regression model was used to obtain the effect of sleep quality, an intermediary effect test was conducted on mental health to further explore the impact of residential perception environment on sleep quality. As shown in Table 10, the mediating benefit test of mental health on the physical environment and sleep quality of the residential area is carried out. Mental health can significantly mediate environmental quality, service facilities, and architectural characteristics, the effect ratios of which are 10.82, 11.93, and 24.17%, respectively. Regarding the public mental health level, sleep quality plays a relatively significant intermediary role in the physical environment of residential areas.

**Table 10:** Summary of test results of physical environment through mental health mediation.

**Item**	**C** **total effect**	***a***	***b***	a^*^b **mediating effect size**	a^*^b **(*p*-value)**	a^*^b **(95% BootCI)**	***c*'** **direct effect**	**Effect ratio**	**Test results**
Environmental quality = > positive mental health = > sleep quality	0.225^**^	0.188^**^	0.130^**^	0.024	0.070	0.012–0.065	0.201^**^	10.820%	Partial intermediary
Vegetation greening = > positive mental health = > sleep quality	0.022	0.111^**^	0.130^**^	0.014	0.155	0.007–0.046	0.007	100%	Fully mediated
Artistic features = > positive mentalh = > sleep quality	−0.021	0.140^**^	0.130^**^	0.018	0.093	0.011–0.053	−0.039	100%	Fully mediated
Architectural characteristics = > positive mental health = > sleep quality	−0.048^*^	0.118^**^	0.130^**^	0.015	0.125	0.009–0.047	−0.063^**^	24.172%	Masking effect
Design scale = > positive mental health = > sleep quality	0.050^*^	0.218^**^	0.130^**^	0.028	0.139	0.019–0.092	0.022	100%	Fully mediated
Service facilities = > positive mental health = > sleep quality	0.193^**^	0.178^**^	0.130^**^	0.023	0.051	0.012–0.058	0.170^**^	11.938%	Partial intermediary

* p < 0.05,

** p < 0.01.

In addition, the intermediary benefits of positive mental health in the social environment and sleep quality of residential areas were tested again as shown in Table 11. In the influence path of positive mental health on residents' sleep quality in the social environment, mental health can have a significant intermediary effect on sense of attachment and safety. The effect accounts for 33.88 and 23.76%, respectively. Compared with the above mediating effects of positive mental health on the impact of the physical environment on residents' sleep quality, the mediating effect of positive mental health level on the impact of social environment on residents' sleep quality is more significant. These are the complete answers to Research Question 3 (RQ3).

**Table 11:** Summary of the test results of social environment through the mediating effect of mental health.

**Item**	***C*** **total effect**	***A***	***b***	a^*^b **mediating effect size**	a^*^b **(*p*-value)**	a^*^b **(95% BootCI)**	***c*'** **direct effect**	**Effect ratio**	**Test results**
Sense of participation = > positive mental health = > sleep quality	0.087^*^	0.269^**^	0.175^**^	0.047	0.002	0.028–0.089	0.040	100%	Fully mediated
Sense of attachment = > positive mental health = > sleep quality	0.125^**^	0.227^**^	0.175^**^	0.040	0.013	0.021–0.085	0.085^*^	31.881%	Partial intermediary
Sense of safety = > positive mental health = > sleep quality	0.165^**^	0.224^**^	0.175^**^	0.039	0.013	0.020–0.081	0.126^**^	23.763%	Partial intermediary

* p < 0.05,

** p < 0.01.

## 4. Discussion

The results show significant differences in sleep quality among residents living in different densities and floors. After exploring the physical and social perceptions of the factors of their residential environment, we built a multiple linear regression model. The environmental quality and service facilities significantly impact sleep quality, and the sense of safety in social environment and sleep quality also have a significant positive correlation. The quality of sleep in the urban high-density residential environment is not high. A higher green space rate of a residential area is not highly beneficial for the current high-density urban living environment. For community renovation, the residential design must reduce the negative impact of a high-density environment, improve residents' quality of living, and enhance social wellbeing. Therefore, the urban settlement model advocated by theories such as new urbanism and compact city still needs to be studied.

### 4.1. Optimize the quality of physical environment and improve sleep quality

The most important way to improve environmental quality is to design road traffic in the residential area in a clean and tidy manner, park the vehicles orderly, and improve the bright lighting at night. Among the multiple regression models, environmental quality has the most significant impact on sleep quality. Road cleanliness and disordered vehicle parking directly increase the “discomfort” at the psychological level. Night lighting has a strong relationship with residents' sense of safety when passing at night. The environmental quality may make the residents easily adapt to the environmental “diffraction” in their psychological state and emotions when sleeping, thus affecting their sleep quality (63).

On the other hand, the residential environment's service facilities carry the residents' activities to a greater extent and provide them with space for leisure, which is an environmental element with a “healing” function. The activities can relieve the pressure and negative emotions of the residents and make them have a relaxed physical and mental state when sleeping. The lack of activity space and service facilities may increase the possibility of sleep disorders (64). Therefore, service facilities are the “catalyst” to improve sleep quality. When building and transforming urban residential areas, we should emphasize the “restorative” leisure environment designs and facilities and the number of service facilities that cannot be ignored.

### 4.2. Create a social environment to reduce sleep disorders

The sense of safety significantly impacts residents' sleep in the social environment, which is the same conclusion of the previous study (65). Fear and restlessness may make a person vulnerable to negative thoughts and emotions, thereby increasing the interference with sleep quality (66). Residents who lack a sense of safety may be in a negative state of sleep quality in residential areas where they are unfamiliar with each other, distrustful, and have a poor living environment (e.g., messy roads and no street lights at night) (67). Therefore, we should strengthen the defensive image of the residential area, enhance the residents' sense of safety, and reduce the probability of residents' sleep disorders.

### 4.3. Improved mental health and enhanced pathways to influence

Previous studies have explored the effects of environment on mental health (68), and the relationship between environment and sleep quality (69, 70). This study further confirms the relationship among residential environment, residents' mental health, and sleep quality through the effect test of the intermediary model. The environment can affect the sleep quality through the residents' mental health. By optimizing the space environment design and improving the social atmosphere of the residential area, not only the sleep quality of the residents can be improved but also the mental health can be effectively promoted (71). This finding deserves the attention of urban planning managers and policymakers, considering the potential impact of urban housing planning and design on residents' sleep quality and their health.

### 4.4. Limitations and prospects

One of the limitations of this study is that there is no further quantification of the development intensity of urban settlements. The residential density is expressed through a plot ratio of settlements, which may affect the generality of the study. On the other hand, the cross-sectional design does not allow us to draw causal relationships and hence future analyses of longitudinal data are required to validate the findings. The findings add to the research on the regulatory effect of human settlements on residents' sleep health. Furthermore, it further emphasizes the importance of considering the residents' psychological state and the social atmosphere of the residential area as the entry point of residents' sleep intervention.

## 5. Conclusion

Based on the subjective questionnaire survey of sleep quality and perceived residential environment, this study explored the different impacts of urban residential environments on sleep quality. According to our findings, several conclusions can be drawn.

First, this quantitative cross-sectional study found that at the macro level, the residents' sleep quality reflected by the residential density and the number of residential floors was significantly different. The urban residential environment is closely related to the sleep quality of residents. Specifically, there are significant differences between the density of urban residential areas and the sleep level of residents. The sleeping level of high-density residential areas (FAR > 2) will be lower. Second, the residential area's physical and social environments significantly correlate with sleep quality to varying degrees, with a considerable level of *P* < 0.01. The most important feature of the environmental quality is 0.333 units of residents' sleep quality, which will be improved with each increase in the perceived level of environmental quality. The most significant factor of a social environment is the sense of safety; every increase in the level of sense of safety will improve the residents' sleep quality by 0.105 units. Finally, we confirmed that residents' mental health significantly mediates the residential environment and the residents' sleep quality.

The study used environmental perceptions and self-report to examine the relationship between the residential environment and sleep. The results provide evidence of human settlements' regulatory role in residents' sleep health. When designing sleep promotion interventions, it is essential to help the public find a balance between improving sleep levels and urban residential space. It further emphasizes the importance of considering the residents' psychological state and the social atmosphere of the residential area.

## Data availability statement

The original contributions presented in the study are included in the article/supplementary material, further inquiries can be directed to the corresponding author.

## Ethics statement

Ethical review and approval was not required for the study on human participants in accordance with the local legislation and institutional requirements. The patients/participants provided their written informed consent to participate in this study.

## Author contributions

Conceptualization and writing—review and editing: XZ and MG. Methodology, software, writing—original draft preparation, and investigation: MG. Validation: XZ, MG, and XC. Formal analysis and data curation: XC. Resources and supervision: XZ. Project administration and funding acquisition: XZ and WZ. Visualization: MG and XC. All authors have read and agreed to the published version of the manuscript.
